# Antileishmanial Activity of Liposomal Clarithromycin against *Leishmania Major* Promastigotes

**Published:** 2012

**Authors:** Ameneh Sazgarnia, Naghmeh Zabolinejad, Pouran Layegh, Omid Rajabi, Fariba Berenji, Zari Javidi, Roshanak Salari

**Affiliations:** 1 *Department and Research Centre of Medical Physics, Avicenna Research Institute, Mashhad University of Medical Sciences.*; 2 *Dermatology, Research Centre for Cutaneous Leishmaniasis, Qaem Hospital, Mashhad University of Medical Sciences, Mashhad, Iran*; 3 *Department of Medicinal Chemistry. Mashhad University of Medical Sciences, Mashhad, Iran*; 4 *Parasitology, Emam Reza Hospital, Mashhad University of Medical Sciences, Mashhad, Iran*; 5 *Dermatology, Research Centre for Cutaneous Leishmaniasis, Emam Reza Hospital, Mashhad University of Medical Sciences, Mashhad, Iran*; 6 *Department and of Pharmaceutical Control, Mashhad University of Medical Sciences, Mashhad, Iran*

**Keywords:** Clarithromycin, * Leishmania major*, Liposome, Promastigote

## Abstract

**Objective(s):**

Cutaneous leishmaniasis is a common parasitic disease which is endemic in some parts of the world. *In vitro* and *in vivo* studies have shown azithromycin efficacy on some *Leishmania* species. Because of structural similarity between clarithromycin and azithromycin and efficacy of clarithromycin against intracellular organisms and due to the absence of previous studies in this respect, we decided to evaluate the efficacy of clarithromycin against promastigotes of *L. major in vitro*.

**Materials and Method:**

First, liposomal and non- liposomal clarithromycin were prepared, then both forms of the drug were incubated with promastigotes for 24 hr in NNN culture media without red phenol in the presence of 5% FCS with different concentrations as follows: 20, 40, 80, 100, 200 and 500 µg/ml.

**Results:**

According to the results, clarithromycin in both liposomal and non- liposomal forms has *in vitro* activity against the promastigotes of *L. major*. The concentration of drug that killed 50% of parasites (ED 50) was 169 and 253.6 µg/ml for liposomal and non- liposomal forms, respectively which shows that lower concentrations of liposomal drug are required to have the same effect as non- liposomal drug and the liposomal form of the drug is more effective than non- liposomal form.

**Conclusion:**

Clarithromycin in both liposomal and non- liposomal forms has *in vitro* activity against the promastigotes of *L. major*.

## Introduction

Cutaneous leishmaniasis (CL) is a chronic protozoal disease that is endemic in many parts of the world. Khorasan state in Northeast Iran is one of the most important areas of CL especially due to *Leishmania tropica *(1). The basis of CL treatment is performed by pentavalent antimoniate compounds, such as systemic and intralesional injection of meglumine antimoniate (Glucantime ^TM^) or sodium stibogluconate (Pentostam) which have many undesired side effects and must be used for several weeks (1, 2). So considering new treatment methods to be effective and to have the least side effects, low cost and easy administration are introduced as interesting procedures for researchers. 

 Azithromycin is one of the macrolide derivatives structurally related to erythromycin and is an appropriate drug for treatment of infectious diseases due to its quick transmission into intracellular compartments, slow release (a half life of 2-6 days) and accumulation in different organs and tissues in high concentrations especially phagocytic cells (2-4). Also its anti-leishmaniasis activity has been reported in some *in vivo* and *in vitro* studies (3).

 Clarithromycin is a semi-synthetic antibiotic from macrolide derivative family (5, 6). Besides its good distribution, it also offers excellent activity against intracellular pathogens such as legionella and *Toxoplasma gondii.* It is effective in cutaneous infections caused by *Mycobacterium cheloni*, *M. simine*, *M. kansasii*, *M. intracellulare*, erythrasma and especially for leprosy treatment (7-10).

 Liposomes are hollow spheres of lipid bilayers, which mainly consist of phospholipids and are widely used as carriers of active ingredients to human tissues and also as lipid transfer vesicles to the skin (11).

 With respect to similar mechanism of action and pharmacokinetic properties of clarithromycin and azithromycin, reports of effective therapeutic anti-leishmaniasis effect of azithromycin and also considering the higher efficacy of liposomal drugs, we decided to evaluate the efficacy of liposomal clarithromycin and compare it with non-liposomal form against *L. major *promastigotes *in vitro*. 

## Material and Methods

***Liposomal preparation***

Liposomal containing clarithromycin were prepared by the dehydration rehydration vesicle (DRV) method (12). The lipid phase containing L-α-phosphatidylcholine (20 µmol/ml) and cholesterol (2:1) were solved in 50 ml chloroform: methanol (2:1, V/V) in a round bottom flask. A thin layer of lipid film was prepared by removing the solvent by rotary evaporator (Buchii, Switzerland). Next, they were freeze-dried (Heto Drywinner, Denmark) overnight to ensure total removal of the solvent. The aqueous phase (50 ml) consisting of clarithromycin (25 µmol/ml) and glucose (250 mM) in phosphate buffer (0.05 M, pH=6.3) was introduced to the total liquid film using vortex at 37°C to achieve a homogenous mixture. 

 The encapsulation percentage of the liposome was found 78% by UV spectroscopic method indirectly.

Stock solution of Clarithromycin (non-liposomal form) was prepared by dissolving 1.8 mg of clarithromycin in 100 ml sterile water for injection.

***Promastigotes cultivation***

*L. major *parasites (*MRHO/IR/75/ER*) gift from Research Centre of Skin Disease and Leprosy, Tehran University of Medical Sciences, kept by passage in BALB/c mice were used for experiments. In order to parasite proliferation, amastigotes from mouse's spleen were cultured in Novy-MacNeal-Nicolle (NNN) medium containing Agar (4 mg/100 ml) and defibrinated rabbit blood (10%). After transformation of amastigotes to promastigotes and their existence from the infected spleen cells, the promastigotes were cultivated in RPMI 1640 (Himedia; AT 028) containing 100 units/mL penicillin, streptomycin 100 μg/mL and 20% FCS in a 27°C incubatior. For parasite passage, flask medium was excluded at first and semi volume of culture was added to it. These passage parasites are usable after 6 days of being kept in incubator, which in this stage were in stationary phase of growth (13). In this step the promastigotes were subjected to the designed experiments.

***Assessment of the drugs effectiveness against promastigotes***

Promastigotes suspension in density of 1× 10 ^7^ parasite/ml were incubated for 24 hr in the presence of different concentrations of liposomal and non- liposomal clarithromycin and phenol red free RPMI culture medium supplemented with 5% FCS. After two times washing, the promastigotes were transferred to 96 well culture plates as 1×10 ^6^ parasite/200 λ in each well and parasite survival was determined by Alamar blue assay. Alamar blue (Biosource, USA) was reduced by promastigotes of *L. major *in a time-dependent process. Optical density of the samples was read by a micro plate reader (Awareness 14; model 3200). Before and 24 hr after adding Alamar blue (20 μl), the absorbance of the samples and medium blank at 545 nm and 630 nm was measured. Absorbance in the absence of drug was set as the 100% control. 

 In this study, we considered concentrations of 20, 40, 80, 100, 200 and 500 µg/ml for both liposomal and non- liposomal clarithromycin in separate groups. In all experiments a control group without parasites incubating with drugs was considered, too. 

***Statistical analysis***

Experiments were repeated at least three times. After calculating parasites survival percentage, SPSS software, version 11.5 (SPSS Inc., Chicago, IL, USA) was used to compare the results in all statistical procedures. Results were expressed as the mean ± SD, and statistical significance was determined by χ2 test; *P* <0.05 was considered significant. ED_50 _was calculated by linear regression analysis or linear interpolation for both drugs_._

## Results

**Figure 1:**
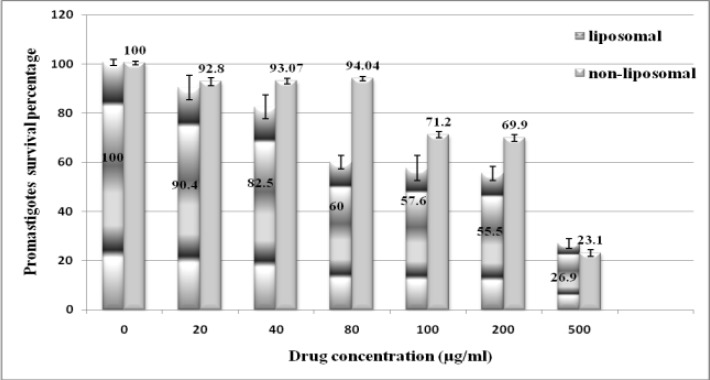
Mean of promastigotes survival percentages after incubating with liposomal and non-liposomal form of clarithromycin. The data represent mean ± SD of the three performed experiments.

Descriptive evaluations of different liposomal and non-liposomal clarithromycin doses are presented in Figure 1, showing that the mean percentage of promastigotes survival decreases with increasing clarithromycin concentrationin of both liposomal and non- liposomal forms. This Figure shows that 80 µg/ml is a critical point in the promastigotes survival curve due to significant decrease of the parasites survival around this concentration in comparison with other ones (*P*<0.05).

 For non-liposomal form of clarithromycin, as it has been shown in Figure 1, 100 µg/ml could be considered as a critical point in decreasing promastigotes survival (*P*<0.05). 

**Figure 2:**
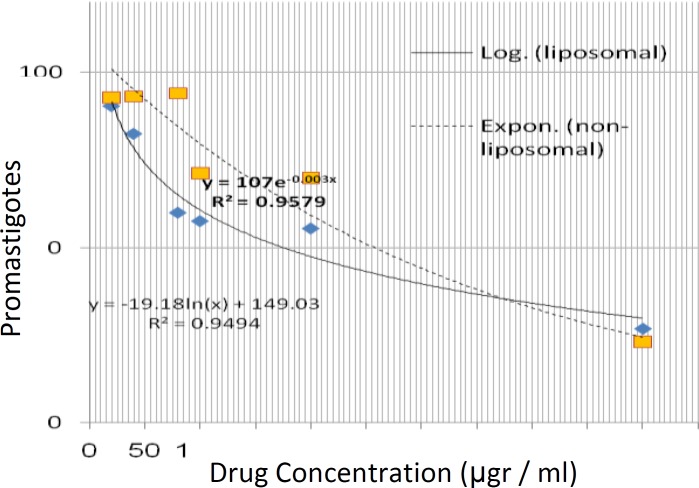
The best fitted mathematical functions on the mean data represented the effectiveness of liposomal and non-liposomal form of clarithromycin against *Leishmania* *major* promastigotes.

 Also, there is a significant difference between lethality induced by liposomal and non-liposomal drugs in the presence of 80 µg/ml clarithromycin to the benefit of liposomal drug (*P*<0.05). Based on Figure 2, the logarithmic and exponential functions were conformed to liposomal and non-liposomal forms. So the half maximum inhibitory concentration (ED_50_) was 169 and 235.6 µg/ml for liposomal and non-liposomal clarithromycin, respectively which means that the lower concentration of liposomal drug is needed for similar therapeutic results than non-liposomal drug. 

## Discussion

In this study clarithromycin was used as liposomal and non-liposomal forms against *L. major in-vitro*. The liposomes are vesicular microscopic forms with phospholipids bilayers that include the aqueous phase. So in order to increase the efficacy, they are used in pharmacodynamic transfer systems for hydrophilic and hydrophobic drugs (14).According to higher efficacy of liposomal drugs, liposomal clarithromycin were used and compared with non-liposomal form. Based on our best knowledge up to now, there is not any similar study of clarithromycin effect against *L. major.*

 Krolewiecki *et al* evaluated the azithromycin efficacy against *L. major in vitro* and *in vivo* (3). They found that ED_50_ of azithromycin on the *L.major* promastigotes was 12 µg/ml. Also Oliveira and others reported *in vitro* antileishmania activity of azithromycin on promastigote and amastigote intracellular cultures against *L.* *amazonensis*, *L.* (Viannia) *braziliensis* and *L.* *chagasi*. They concluded that azithromycin effect for the three species studied has been dose-dependent, which is in accordance with result provided for *L .major *(4).Our results showed that clarithromycin was effective *in vitro* in both liposomal and non-liposomal forms. The ED_50_ for liposomal and non-liposomal clarithromycin forms were 109 and 253.6 µg/ml respectively. So the lower dose was needed for liposomal forms till obtaining the similar results.The mechanism by which clarithromycin decreases the number of parasite remains unclear as for azithromycin (4). Since it is easily diffused into most tissues and phagocytes and due to the high concentration in phagocytes, clarithromycin is actively transported to the site of infection. During active phagocytosis, large concentrationsof clarithromycin are released. The concentration of clarithromycin in the tissues could be 10 times higher than in plasma (14). There are some evidences that suggests the fact that the drug is effective against not only bacteria but also protozoa such as *T. gondii*, *Cryptosporodium *spp. and *Plasmodium *spp (15). Its action mechanism that affects microorganisms is carried out through reversibly connecting to 50S ribosomal subunits and inhibiting the protein synthesis (16). In *in vitro *studies, it has been asserted that clarithromycin does not produce a morphological change on *T. gondii*, but affects it by reducing the number of host cells infected with parasites and the number of parasites in these cells (17-19). So we suggest that, clarithromycin probably produces a direct effect on promastigotes of *L. major *for *T. gondii, *as it that has been previously demonstrated for another family member of macrolids, azithromycin (3, 4).

## Conclusions

This study was the first *in vitro* study of anti-leishmaniasis effects of clarithromycin that showed positive results against *L. major *promastigotes. We recommend the future study on intracellular amastigotes and then on the animal models like infected Balb/c mice. Hopefully the drug development steps could support clinical trial decisions.
